# Supplementary material for: Exploring the Link Between Obligate Anaerobe-Related Dysbiosis and Prostate Cancer Development: A Pilot Study
Source: Cancers (Basel). 2024 Dec 29;17(1):70. doi: 10.3390/cancers17010070 (PMC11720123; doi:10.3390/cancers17010070)
Supplement: Supplementary file 1 [file cancers-17-00070-s001.zip › supplementary-Figures-Tables-revised-v2.pdf]

Supplementary Files in Excel format

<u>File: "Table S1.xlsx":</u> Read counts with taxonomic annotation per ASV per sample for the first sequencing batch of 50 samples

<u>File: "Table S2.xlsx":</u> Read counts with taxonomic annotation per ASV per sample for the second sequencing batch of 39 samples

Supplementary tables in Word format

Centre	Bacteria	Mean PSA (min – max)	Median PSA	N*
All	Neither	4.75 (0.3-17.6)	3.40	22
All	Microaerophilic	6.51 (2.43-12.0)	6.34	6
All	Obligate anaerobe	9.25 (1.12-26.0)	6.75	12

Table S3. Summary result of PSA vs type of bacteria for Barts and Newham patients. *N=40, Of the 18 patients having anaerobes, 1 had both Obligate anaerobe and Microaerophilic bacteria and actual number of individual patients with either OA &/or MA is 17 out of 39 (43.5%). "Neither": samples with no miroaerophillic or obligate anaerobe species detected

	No of Cases	TUR of Prostate	Cancer interventions	Continued surveillance	PSA median	PSA Mean (range)
Anaerobe negative PC negative	11	1	0	10 (91%)	0.75	2.89 (0.3-17.6)
Anaerobe positive PC negative	7	2	0	5 (71%)	4.54	5.39 (1.1-13.6)
Anaerobe negative PC positive	11	0	4	7 (64%)	6.70	6.61 (1.3-12)
Anaerobe positive PC positive	10	1	7	2 (20%)	7.36	10.49 (2.4-26)

Table S4. Long term follow-up of pooled analysis of anaerobe* verses Prostate Cancer and PSA. *Anaerobe positive status includes both obligate anaerobes and microaerophilic

	OA positive	OA negative	OA threshold
Culturomics cohort (N=39)	30.8% (n=12)	69.2% (n=27)	-
PSA (mean ng/mL)	9.25 (p=0.132)	5.04	-
PROVENT series (N=89)	29.2% (n=26)	70.8% (n=63)	≥10% of all genera
PSA (mean ng/mL)	7.65 (p=0.747)	7.38	≥10% of all genera
PROVENT series (N=89)	25.8% (n=23)	74.2% (n=66)	≥15% of all genera
PSA (mean ng/mL)	8.13 (p=0.291)	7.23	≥15% of all genera
PROVENT series (N=89)	19.1% (n=17)	80.9% (n=72)	≥20% of all genera
PSA (mean ug/L)	9.30 (p=0.007)	7.03	≥20% of all genera

Table S5. Sample frequency and mean PSA for OA positive and OA negative groups. Culturomics samples were stratified based on the presence of one or more OAs. The nine OA genera used to stratify PROVENT samples are: Finegoldia, Fusobacterium, Prevotella, Peptoniphilus_A, Peptoniphilus_B, Peptoniphilus_C, Peptostreptococcus, Veillonella and Veillonella_A. OA threshold: Pooled relative abundance threshold for a PROVENT sample to be considered as OA positive. The displayed p-value is from Welch's two-sample t-test

	Number of Positive Patients	Median Relative Abundance (+/- of those >0%)	Percentage ≥10%/Number Relative Abundance
Ochrobactrum_A	n = 14	0.084%<br>0.016-0.803%	n = 0/14 (0%)
Fusobacterium	n = 10	0.199%<br>0.006-41.25%	n = 2/10 (20%)
Peptostreptoccus	n = 9	0.718%<br>0.009 – 3.703%	n = 0/9 (0%)
Peptoniphilus_A	n = 43	0.950%<br>0.002-69.83%	n = 1/43 (2.3%)
Prevotella	n = 44	5.597%<br>0.007-62.73%	n = 17/44 (38.6%)

Table S6. Relative abundance of 5 bacteria of interest in PROVENT series (n=89)

<u>**Supplementary Figures**</u>

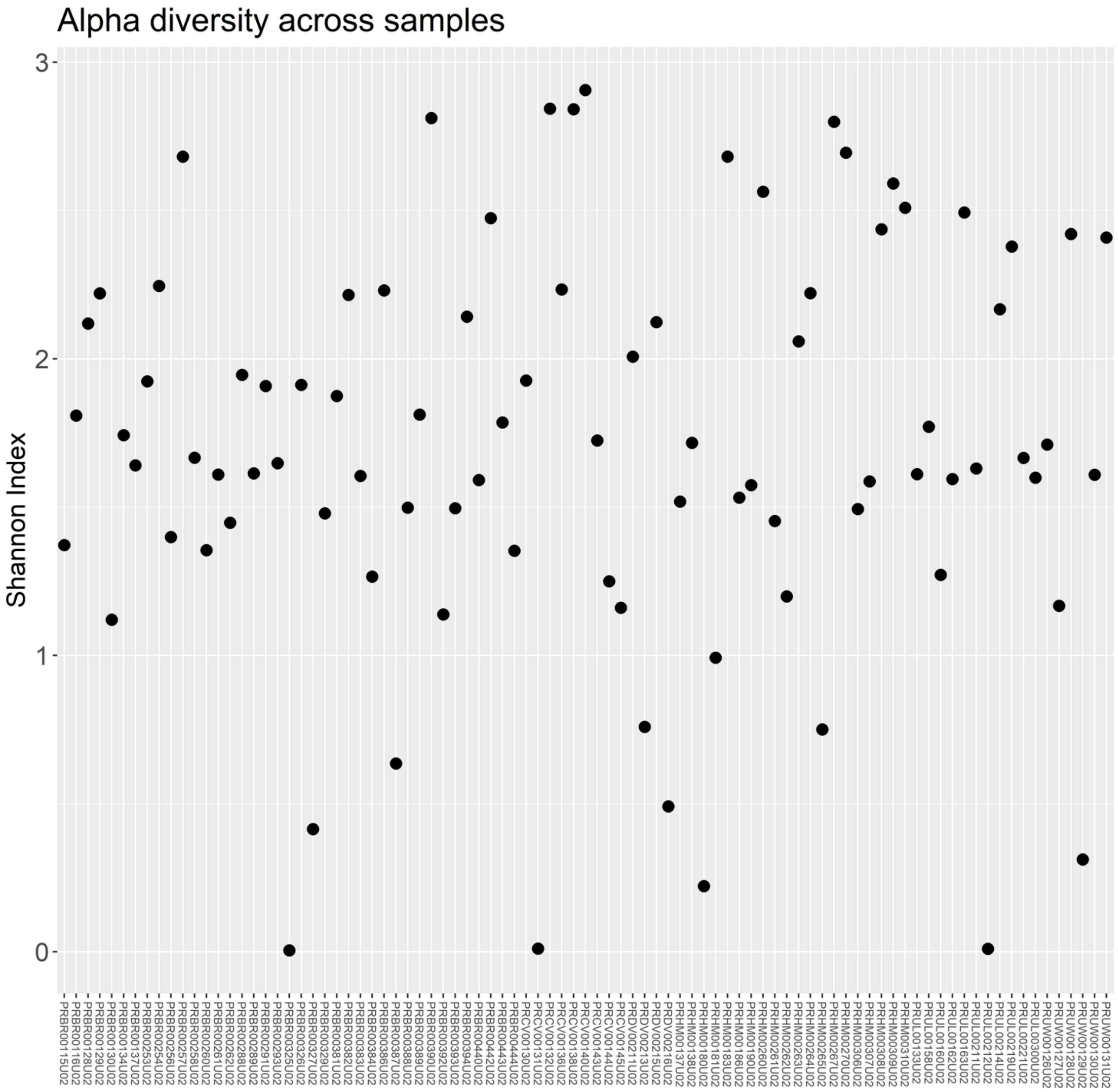

Figure S1. Alpha diversity (Shannon index) per sample from the PROVENT cohort (n = 89). Higher Shannon index values indicate greater microbial diversity.

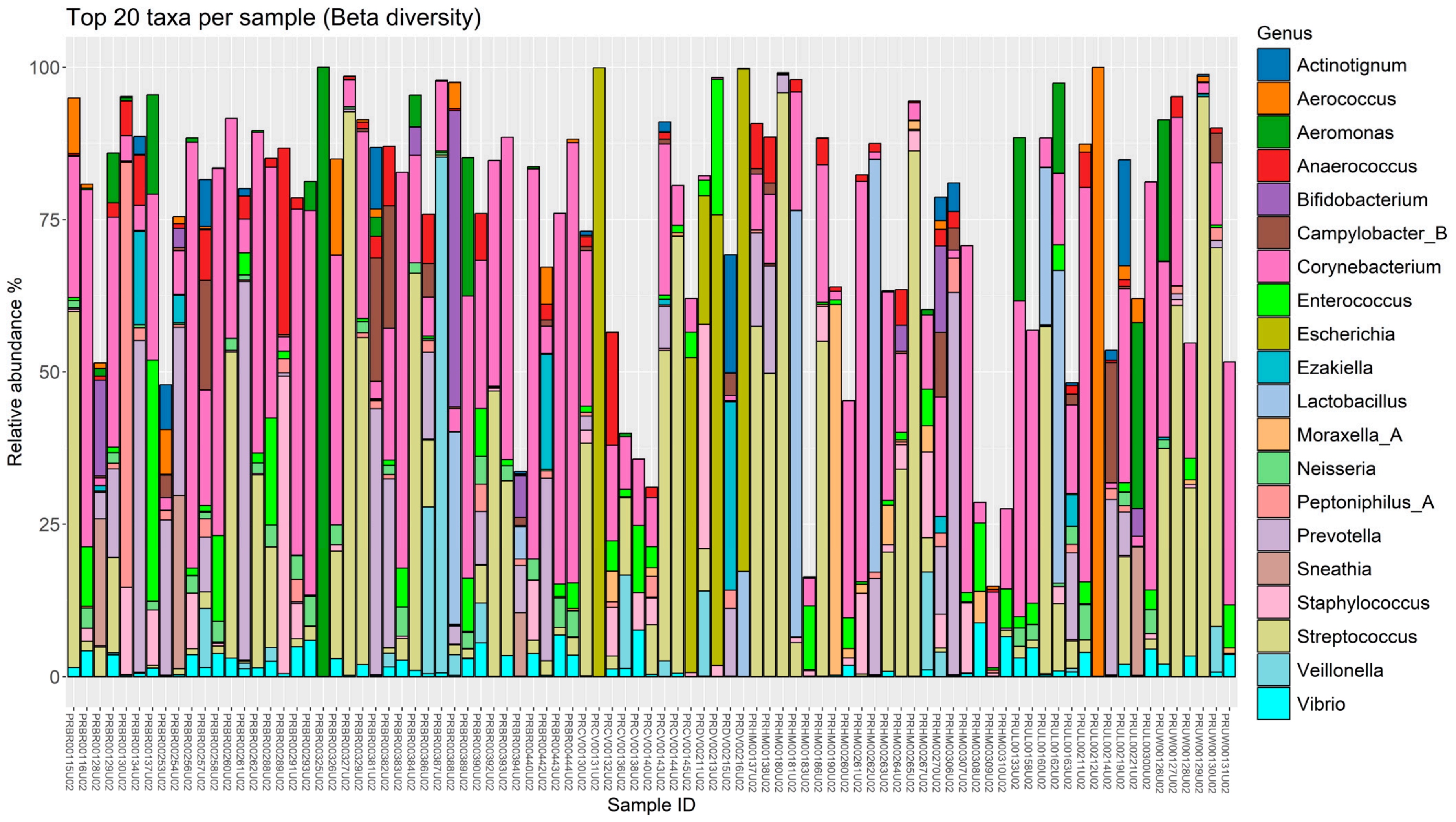

Figure S2. Beta diversity representation of the samples from the PROVENT cohort (n = 89) using the 20 most abundant genera. Samples whose relative abundance does not amount to 100% are due to the exclusion of unidentified ASVs to the Genus level as well as due to the exclusion of ASVs not belonging to the 20 most abundant genera.

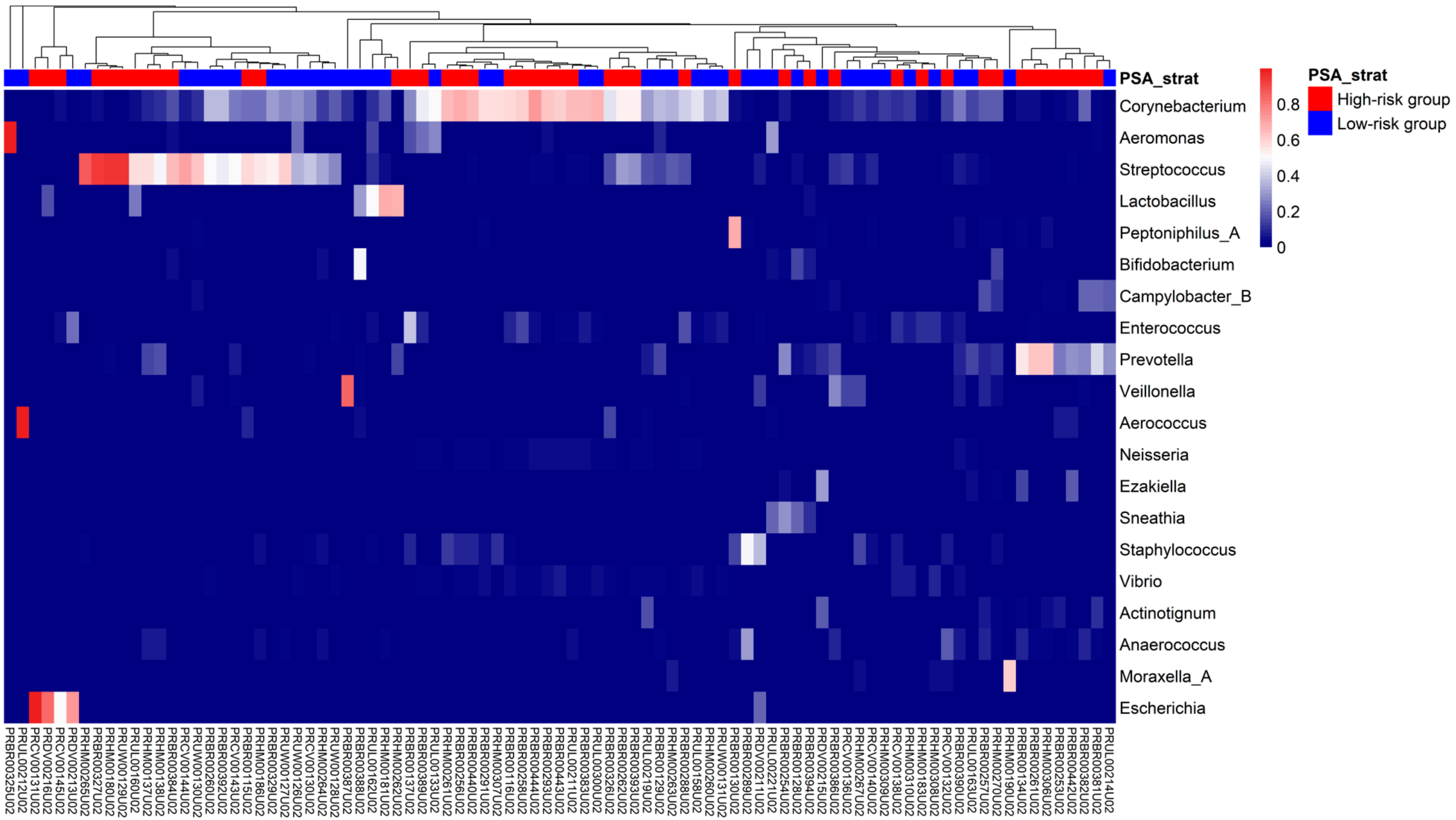

Figure S3. Heatmap displaying the relative abundances (0 = 0%, 1 = 100%) of the top 20 most abundant genera per sample from the PROVENT cohort (n = 89). The color gradient in each rectangle represents the relative abundance of a particular genus (row) in a particular sample (column): darker red shades = higher abundances, darker blue shades lower abundances. PSA_strat: color code of high/low risk groups based on median PSA stratification: red = High-risk group (PSA > median PSA), blue: Low-risk group (PSA ≤ median PSA). Hierarchical clustering was used to order samples (columns) based on the relative abundances.

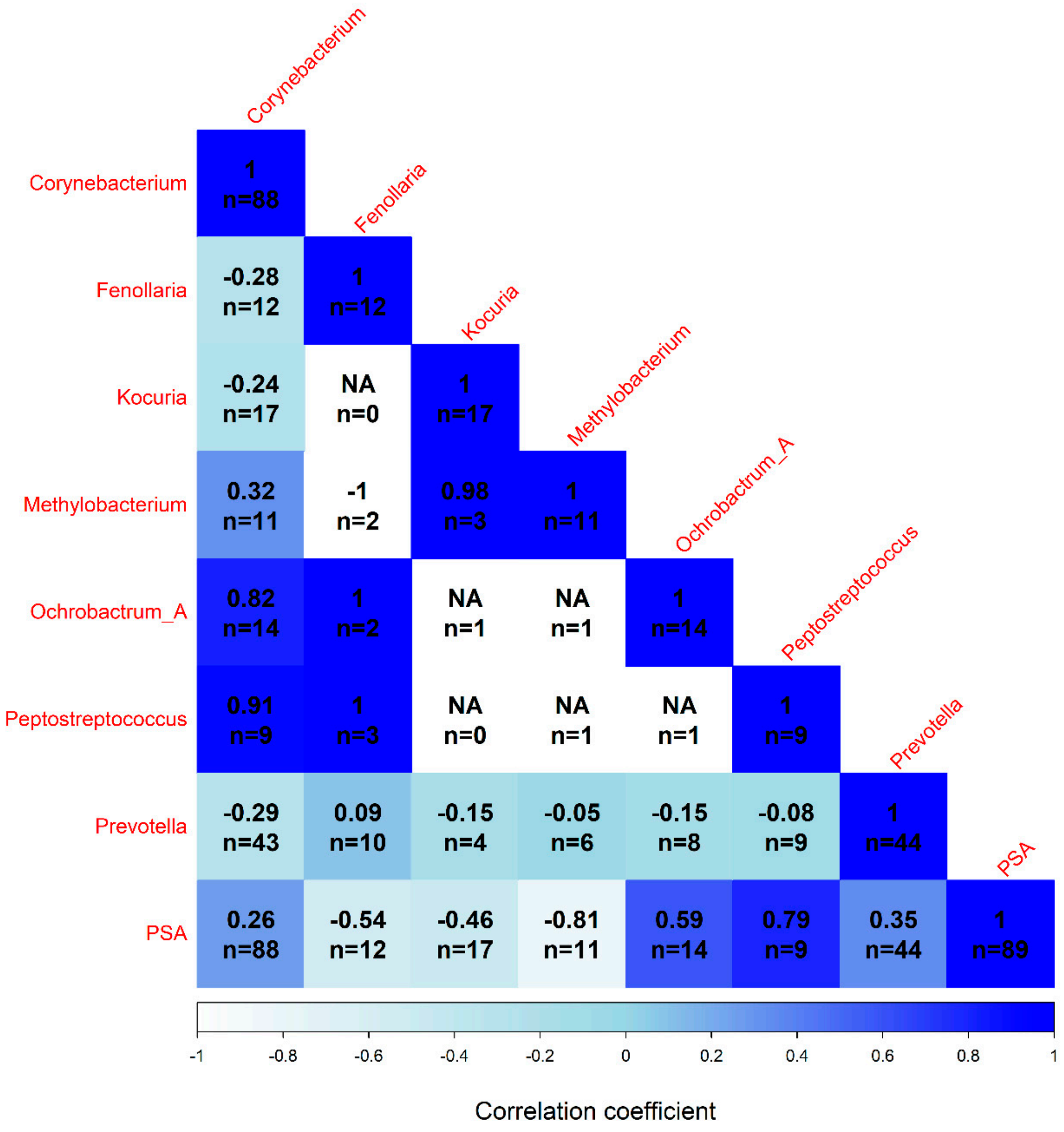

Figure S4 Correlation matrix illustrating the relationships between the relative abundance of genera and PSA levels. All displayed genera had a Pearson correlation with PSA with a p-value < 0.1. The Pearson correlation coefficient and the number of samples taken for each comparison is displayed in each cell. NA: correlation coefficient could not be estimated due to lack of an appropriate number of samples.
