# Supplementary figures and images for: Exploring the Link Between Obligate Anaerobe-Related Dysbiosis and Prostate Cancer Development: A Pilot Study
Source: Cancers (Basel). 2024 Dec 29;17(1):70. doi: 10.3390/cancers17010070 (PMC11720123; doi:10.3390/cancers17010070)

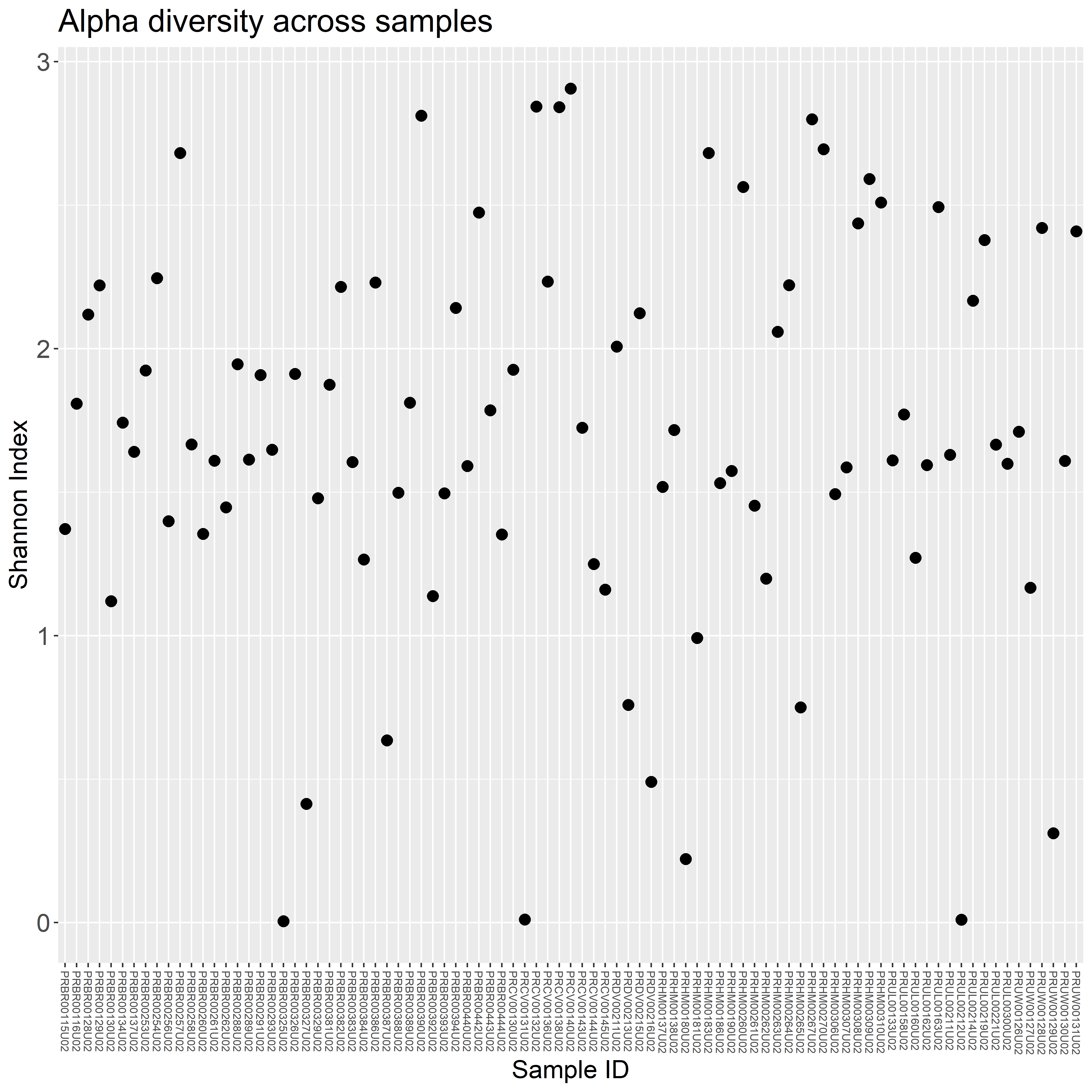

Supplement: Supplementary file 1 [file cancers-17-00070-s001.zip › Figure S1.png]

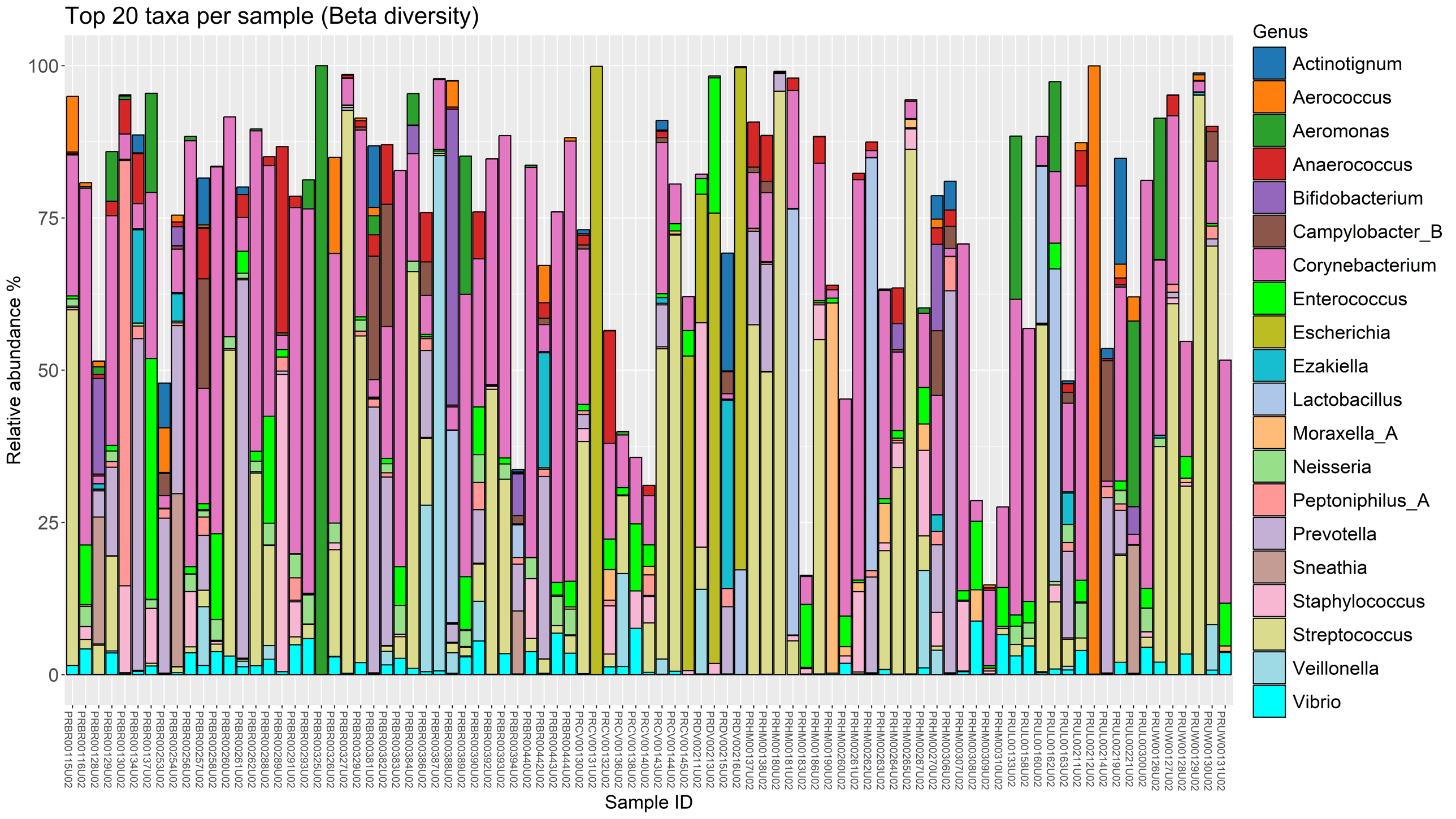

Supplement: Supplementary file 1 [file cancers-17-00070-s001.zip › Figure S2.png]

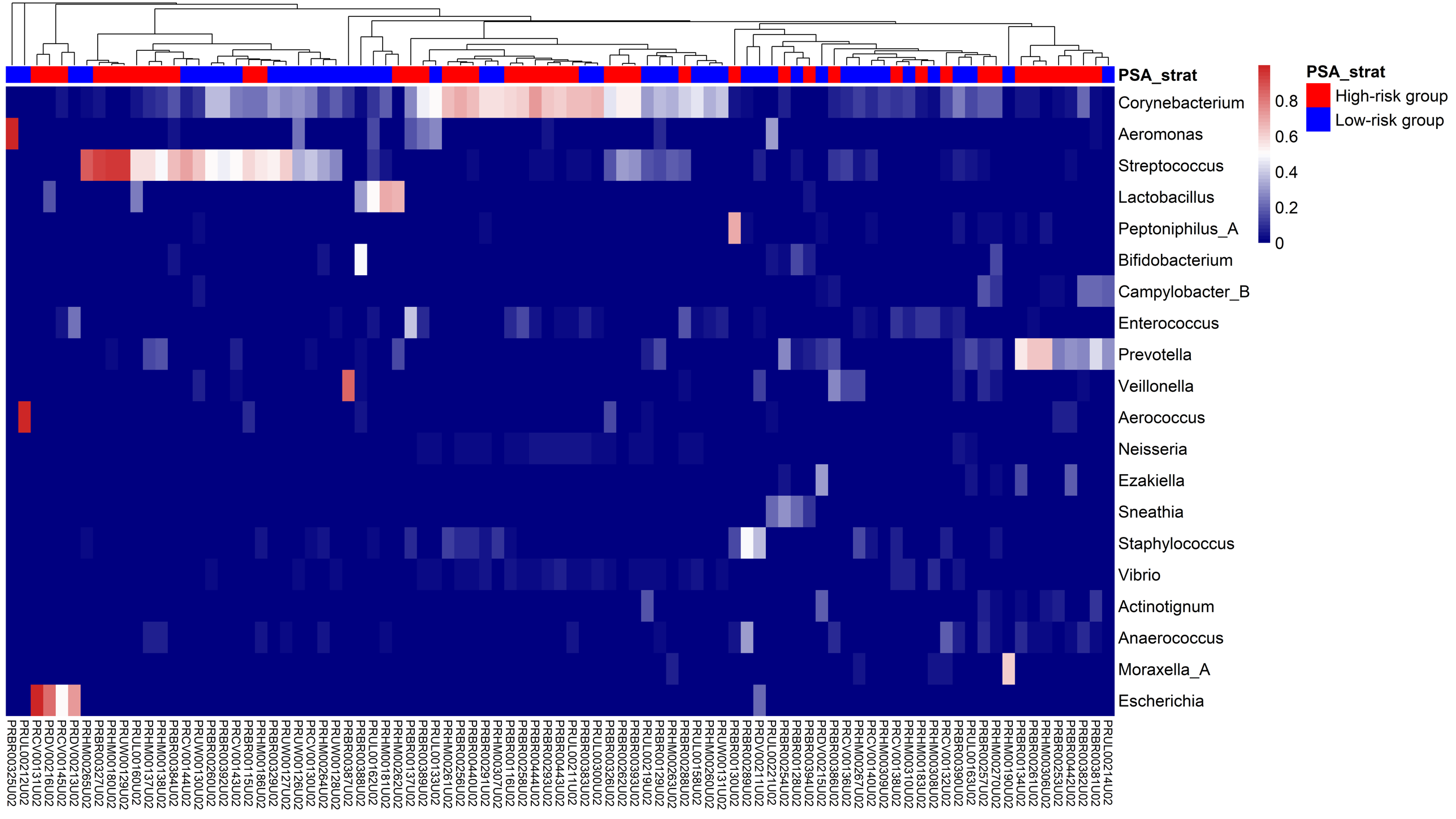

Supplement: Supplementary file 1 [file cancers-17-00070-s001.zip › Figure S3.png]

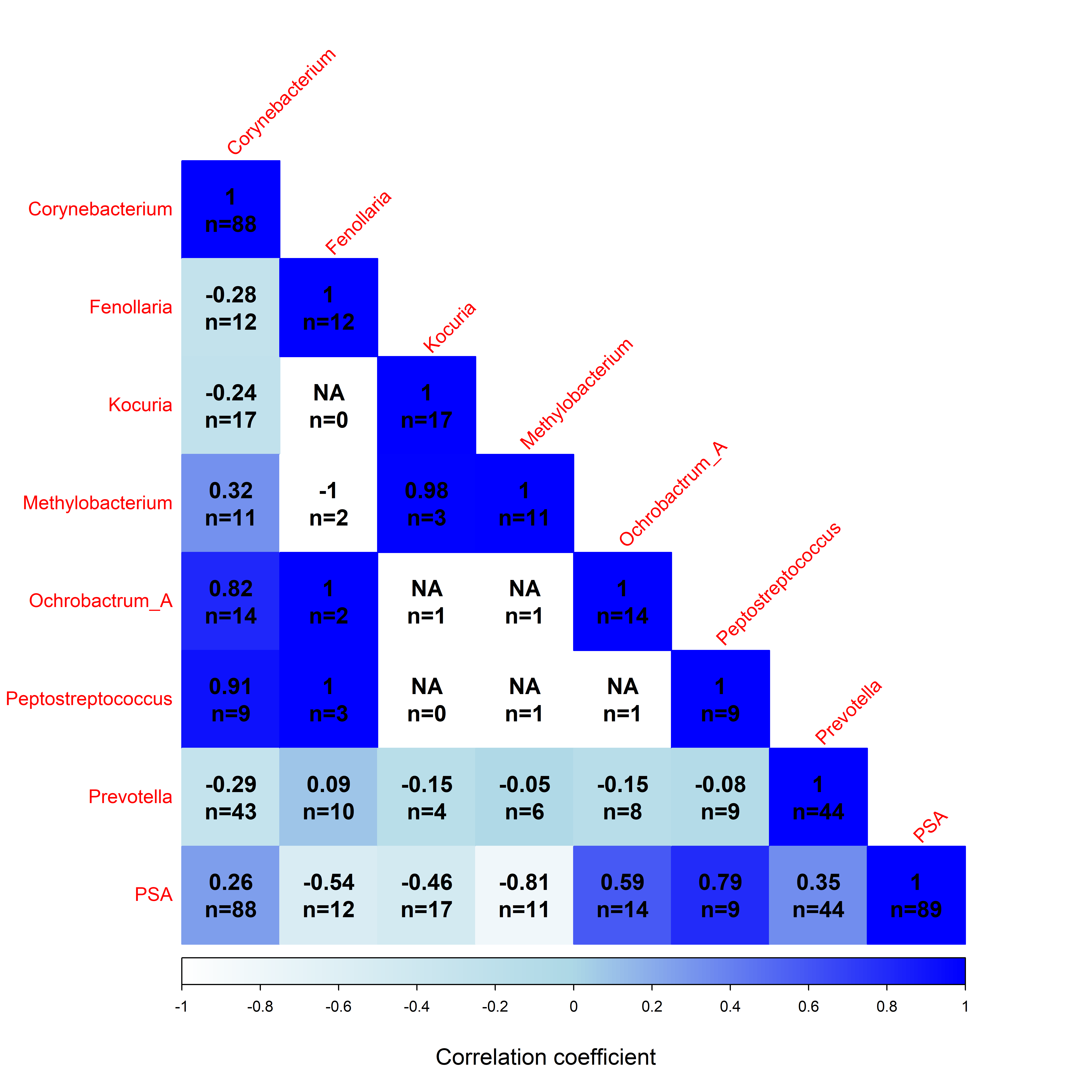

Supplement: Supplementary file 1 [file cancers-17-00070-s001.zip › Figure S4.png]
